# Multimodal Imaging of Acquired Vitelliform Lesion Diagnosed at Pseudohypopyon Stage

**DOI:** 10.1155/2013/461758

**Published:** 2013-04-24

**Authors:** Nuno Moreira Gonçalves, Ângela M. Carneiro, Elisete Brandão, Fernando M. Falcão-Reis

**Affiliations:** ^1^Department of Ophthalmology, Hospital São João, Alameda Prof. Hernâni Monteiro, 4200-319 Porto, Portugal; ^2^Department of Sense Organs, Faculty of Medicine, University of Porto, Portugal

## Abstract

*Purpose*. To present a case study of a monocular acquired vitelliform lesion, studied with multimodal fundus imaging (spectral-domain-optical coherence tomography, fundus autofluorescence, and fluorescein angiography) with a followup of three years. *Case Report*. An asymptomatic macular lesion was detected on a 64-year-old man. Fundus exam revealed a macular lesion with an apparent horizontal level associated with multiple round small whitish lesions, suggestive of cuticular drusen. He was studied with autofluorescence of the fundus (FAF), fluorescein angiography (FA), spectral domain-optical coherence tomography (SD-OCT), and electrooculogram. The findings were compatible with the diagnosis of acquired vitelliform lesion, associated with cuticular drusen. After one year, the visual acuity decreased to 20/50, without identifiable alterations of the FAF, FA, or SD-OCT. Three years later, fundoscopy and imaging showed an evolution to a state similar to vitelli disruptive phase of Best disease with an improvement of visual acuity to 20/25. We report the results of FAF, FA, and SD-OCT at this stage. *Conclusion*. Acquired vitelliform lesions associated with cuticular drusen can present as a pseudohypopyon lesion, and the evolution to the atrophic phase can be associated with an improvement of visual acuity.

## 1. Introduction

The term vitelliform lesions refers to accumulation of yellowish subretinal material. In younger patients, they usually occur in the setting of Best vitelliform macular dystrophy, an autosomal dominant disorder, associated with mutations in bestrophin 1 gene [1].

In adults, vitelliform lesions can occur associated with various disorders: age-related macular degeneration, cuticular drusen, or tractional maculopathies [1].

The classical staging of Best vitelliform macular dystrophy divides the progression of the disease into five stages: subclinical, vitelliform, pseudohypopyon, vitelliruptive, and atrophic [2]. These stages are also observed in acquired lesions; however, the pseudohypopyon stage is rarely identified. The case report we present shows a multimodal image study of an acquired vitelliform lesion, associated with cuticular drusen, diagnosed in the stage of pseudohypopyon.

## 2. Case Presentation

In April 2009, a 64-year-old man was referred to the Retina Department to study a macular lesion OS. There was the suspicion of a choroidal neovascular lesion. The patient had a history of trauma to OD during childhood, with resulting traumatic cataract, exotropia, and amblyopia of OD. He had undergone phacoemulsification with insertion of an anterior-chamber intraocular lens (IOL) in 2007 and a strabismus surgery in 2008.

At the examination, visual acuity was 20/200 OD and 20/25 OS. The slit lamp exam was unremarkable in OS and revealed a well-positioned IOL in OD.

**Figure 1:**
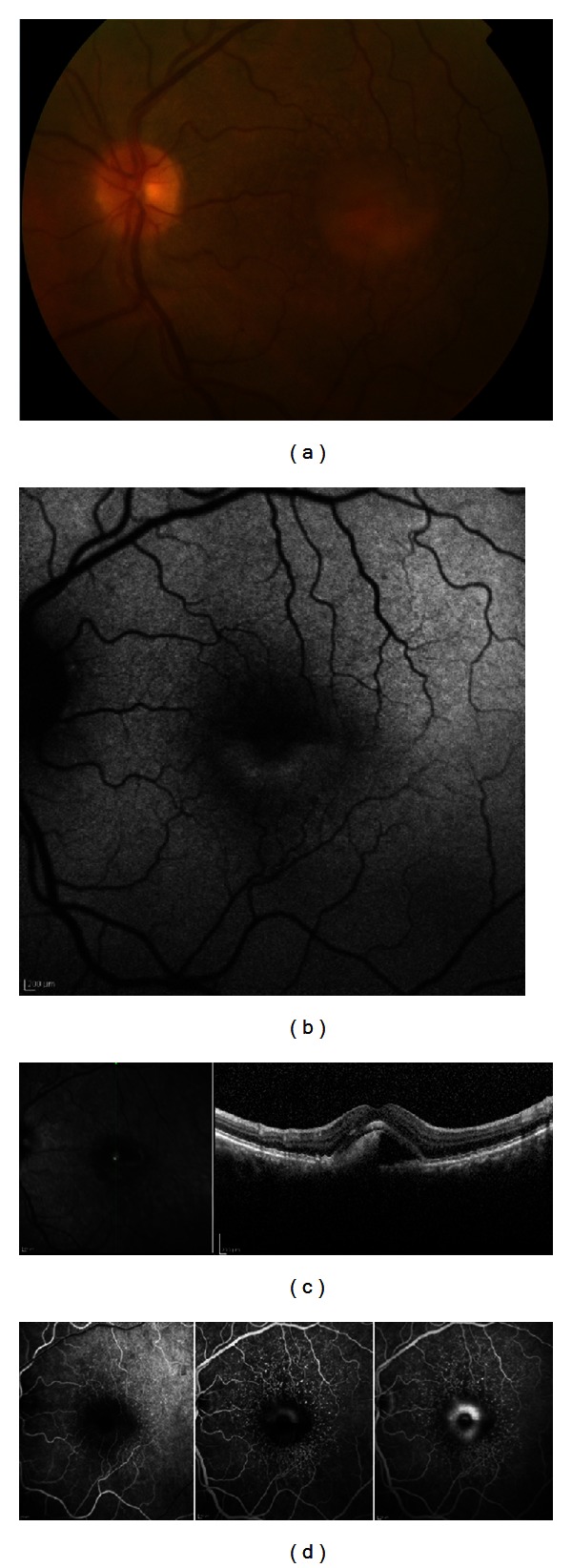
(a) Retinography of the left eye showing a circular yellowish macular lesion with an apparent horizontal level. (b) Fundus auto-fluorescence of the left eye with hyperautofluorescence of the bottom half of the lesion and hypoautofluorescence of the top half. (c) Vertical SD-OCT section of the left eye showing hyperdense subretinal material accumulated in the bottom half. (d) Fluorescein angiography of the left eye with the “stars-in-the-sky” pattern and late hyperfluorescence of the macular lesion.

Fundus exam of OD revealed a myopic choroiditis and a tilted disc. In OS was observed a round yellowish macular lesion, with well-defined limits and an apparent horizontal level. There were also multiple round small whitish lesions, suggestive of cuticular drusen (Figure 1(a)).

Autofluorescence of the fundus (FAF) of OS showed multiple small hypoautofluorescent lesions and a macula lesion, divided by a horizontal level, with a hyperautofluorescent bottom half and a hypoautofluorescent superior half (Figure 1(b)).

The high-resolution spectral domain-optical coherence tomography (SD-OCT) showed small elevations of the retinal pigment epithelium (RPE) and an accumulation of hyper-reflective material in the subretinal space, above the RPE, deposited in the inferior half of the lesion (Figure 1(c)). 

The fluorescein angiography (FA) of OS showed early hyperfluorescent lesions, corresponding to the typical “stars-in-sky” pattern of cuticular drusen. The macular lesion showed an early hypofluorescence and a late hyperfluorescence in its bottom half (Figure 1(d)).

The patient performed electrooculogram (EOG) that revealed a normal Arden ratio OU (2.08 OD and 1.99 OS). The electroretinogram (ERG) in photopic and scotopic conditions was also a normal OU. The ERG pattern showed a reduction of the P50 component OU.

The clinical and imaging findings, combined with a normal EOG, were suggestive of the diagnosis of acquired vitelliform lesion, associated with cuticular drusen. It was decided to evaluate the patient periodically.

After one year, the visual acuity of OS decreased to 20/50, without identifiable alterations of the FAF, FA, or SD-OCT. This was attributed to progression of a cataract. In 2011, two years after the first examination, visual acuities were similar. At this time, the decision was to wait one more year before proposing phacoemulsification.

**Figure 2:**
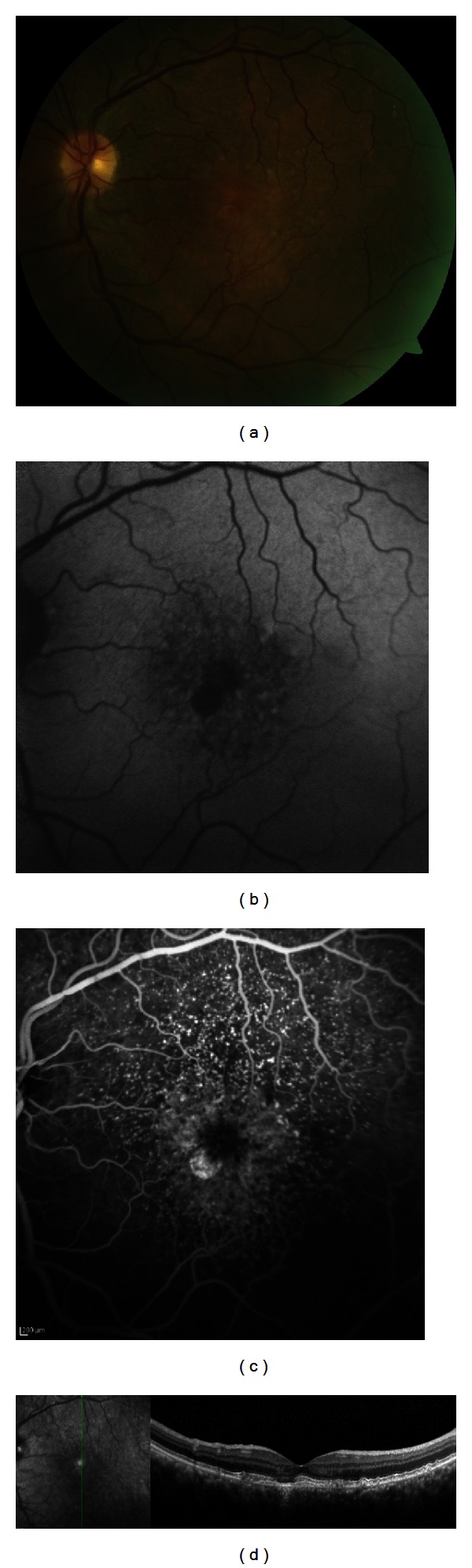
(a) After 3 years of followup, the fundoscopic aspect showed a resolution of the previous lesion. (b) The FAF shows two well-circumscribed areas of hypoautofluorescence. (c) Fluorescein angiography with the “stars-in-the-sky” pattern. (d) Vertical SD-OCT section shows integrity of the IS/OS junction, with an area of RPE atrophy adjacent to the fovea.

In 2012, there was an increase of visual acuity of OS to 20/25. Fundus examination showed disappearance of the previous lesion, with evolution to a state similar to vitelli disruptive phase of Best disease (Figure 2(a)). FAF revealed two well-circumscribed round areas of EPR atrophy (Figure 2(b)) with disappearance of the hyperautofluorescent material. The FA showed the same “stars-in-the-sky” pattern, with hyperfluorescence of the areas of RPE atrophy (Figure 2(c)). SD-OCT revealed that, in the fovea, the EPR was integer as long as the photoreceptor inner/outer segment (IS/OS) junction (Figure 2(d)).

## 3. Discussion

We reported a case of an acquired vitelliform lesion, associated with cuticular drusen and diagnosed in the stage of pseudohypopyon. The evolution is documented with multimodal imaging from this stage to the atrophic phase.

Vitelliform lesions in adult setting have been described associated with different entities, namely, cuticular drusen. This is an association known since some decades ago [3].

Cuticular drusen occur at earlier ages (<50 years old) and have a different angiographic behavior when compared with drusen related to age-related macular degeneration (AMD). These ones usually have late hyperfluorescence, whilst cuticular drusen are hyperfluorescence since the early times of the exam, resulting in the typical “stars-in-the-sky” pattern [4], as represented by our patient's OS FA.

SD-OCT and FAF have important roles in the study of this pathology. It shows the elevation of the RPE in a “sawtooth” pattern, and it locates the macular detachment at the subretinal level, as opposed to the sub-RPE level, typical of RPE detachment in exudative AMD. It is also crucial, when in association with FA, in excluding the presence of a choroidal neovascular membrane. The pattern of multiple, small hypoautofluorescent lesions is very suggestive of the diagnosis of cuticular drusen. In a recent series of cases with cuticular drusen, vitelliform macular detachment has been found in 11.9% of the studied eyes [4].

So, both SD-OCT and AF have an important role in the followup of these patients, detecting alterations in the macular detachment, the subretinal material, and the development of complications.

Regarding the electrophysiologic exams, the normal Arden ratio of EOG in our patient was a primordial factor for excluding Best disease. Nevertheless, his two siblings were also submitted to mydriatic fundoscopy, with no changes being found.

Pseudohypopyon has rarely been described in acquired vitelliform lesions [1]. It represents a stage of deposition of the subretinal material inferiorly in the macular detachment and, in Best disease, it usually precedes the vitelli disruptive phase. In our case, we could document the integrity of the IS/OS junction with SD-OCT during all the followup.

We could not explain the temporary decrease of visual acuity in our case, but, as has been described in literature, the resolution of the lesion is usually spontaneous and can be accompanied by an increase in visual acuity. So, the best course of action in these cases is surveillance, with periodic examination, in order to detect possible complications (like choroidal neovascularization). Regarding other possible therapeutic options, photodynamic therapy has been tried for vitelliform lesions with no effect on visual acuity [5]; intravitreal bevacizumab has proved some efficacy in the treatment of choroidal neovascularization but not of pure vitelliform lesions [6].

This report shows a case of a monocular acquired vitelliform lesion, associated with cuticular drusen, diagnosed in the stage of pseudohypopyon, and described as a rare finding in the literature. The multimodal imaging analysis strokes some light into the identification of structural abnormalities; however, in some particular cases, there is a decrease in the visual function with no distinguishable alteration in the imaging exams.
